# Cement-Augmented Screw Fixation for Unreconstructible Acetabular Posterior Wall Fractures: A Technical Note

**DOI:** 10.3390/life15101573

**Published:** 2025-10-09

**Authors:** Jihyo Hwang, Ho won Lee, Yonghyun Yoon, King Hei Stanley Lam

**Affiliations:** 1 Department of Orthopaedic Surgery, Gangnam Sacred Heart Hospital, Hallym University College of Medicine, Seoul 07441, Republic of Korea; 2 Department of Orthopaedic Surgery, Gangdong Sacred Heart Hospital, Seoul 05355, Republic of Korea; 3 Incheon Terminal Orthopedic Surgery Clinic, Incheon 21574, Republic of Korea; 4 International Academy of Regenerative Medicine, Incheon 21574, Republic of Korea; 5 MSKUS, 1035 E. Vista Way #128, Vista, CA 92084, USA; 6 The Faculty of Medicine, The University of Hong Kong, Pokfulam, Hong Kong; 7 Board of Clinical Research, The International Association of Musculoskeletal Medicine, Kowloon, Hong Kong; 8 The Faculty of Medicine, The Chinese University of Hong Kong, New Territories, Hong Kong

**Keywords:** acetabular fracture, posterior wall, comminuted, cement augmentation, joint preservation, polymethylmethacrylate (PMMA), joint salvage procedure

## Abstract

The management of severely comminuted acetabular posterior wall fractures in young, active patients presents a significant surgical challenge. When anatomical open reduction and internal fixation (ORIF) is not feasible, primary total hip arthroplasty (THA) is often considered but is a suboptimal solution due to concerns over long-term implant survivorship and the inevitability of revision surgery. This single-patient technical note presents a novel joint-preserving technique for managing unreconstructible acetabular posterior wall fractures using with cement-augmented screw fixation via the Kocher–Langenbeck approach. A 28-year-old male sustained a left posterior hip dislocation with a comminuted acetabular posterior wall fracture involving >30% of the articular surface, alongside a tibial shaft fracture, following a high-energy motorcycle collision. Intraoperative assessment confirmed the posterior wall was unreconstructible, with six non-viable osteochondral fragments. A joint-preserving salvage procedure was performed. After debridement, a stable metallic framework was created using three screws anchored in the posterior column. Polymethylmethacrylate (PMMA) bone cement was then applied over this framework in its doughy phase, meticulously contoured to reconstruct the articular surface. The hip was reduced, and the tibia was fixed with an intramedullary nail. The patient was mobilized with weight-bearing as tolerated on postoperative day 3. At the 21-month follow-up, the patient reported no pain during daily activities and only mild discomfort during deep squatting. Radiographic and CT evaluations demonstrated a stable hip joint, concentric reduction, well-maintained joint space, and no evidence of implant loosening or osteolysis. Level of Evidence: V (Technical Note/single-patient Case report). For unreconstructible, comminuted fractures of the non-weight-bearing portion of the acetabular posterior wall in young patients, cement-augmented screw fixation offers a viable joint-preserving alternative to primary THA. This technique provides immediate stability, facilitates early mobilization, and preserves bone stock. While long-term outcomes require further study, this case demonstrates excellent functional and radiographic results at 21 months, presenting a promising new option for managing these complex injuries.

## 1. Introduction

Fractures around the hip joint are broadly categorized into high-energy traumatic fractures and insufficiency fractures in the elderly. While insufficiency fractures, often associated with osteoporosis and sarcopenia, frequently occur in the femur and have established treatment algorithms culminating in arthroplasty if reduction is unsatisfactory, the management of high-energy acetabular fractures in young adults presents a significant surgical challenge [1,2,3,4]. The options are limited, especially when severe comminution and articular cartilage damage preclude anatomical open reduction and internal fixation (ORIF). In such scenarios, primary total hip arthroplasty (THA) is often considered but is a suboptimal solution for young, active patients due to concerns over long-term implant survivorship, activity restrictions, and the inevitability of complex revision surgery [5,6].

Unreconstructible fracture dislocations are orthopedic emergencies. Delay can lead to accelerated soft tissue damage, contractures, and secondary neurovascular compromise [7]. Even with successful reduction, extensive articular surface injury carries a high risk of post-traumatic osteoarthritis, potentially leading to disability and the eventual need for THA [7].

This technical note describes a novel joint-preserving salvage technique for managing unreconstructible, comminuted acetabular posterior wall fractures with significant articular defects. We present the case of a young male patient treated with cement-augmented screw fixation, avoiding primary arthroplasty, and report excellent functional outcomes at a 21-month follow-up.

## 2. Materials and Methods

### 2.1. Patient Information

A 28-year-old Asian male was admitted following a high-energy motorcycle collision. Informed consent was obtained for the procedure and publication of this report.

This retrospective case study involved analysis of completed treatment with de-identified patient data and informed patient consent. Accordingly, it was exempt from institutional review board approval under local guidelines for single-patient technical reports.

### 2.2. Preoperative Assessment

**Figure 1:**
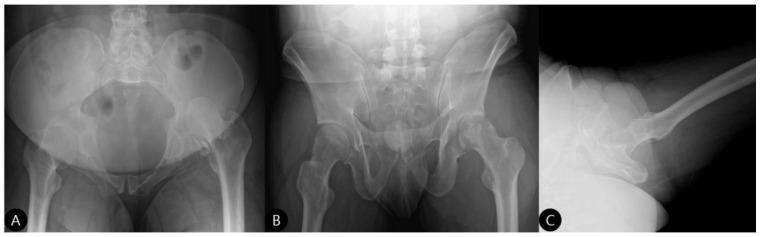
Initial radiographs upon admission (2 October 2023). (**A**) Pelvic outlet, (**B**) inlet, and (**C**) cross-table lateral views demonstrating a left acetabular posterior wall fracture with posterior hip dislocation.

**Figure 2:**
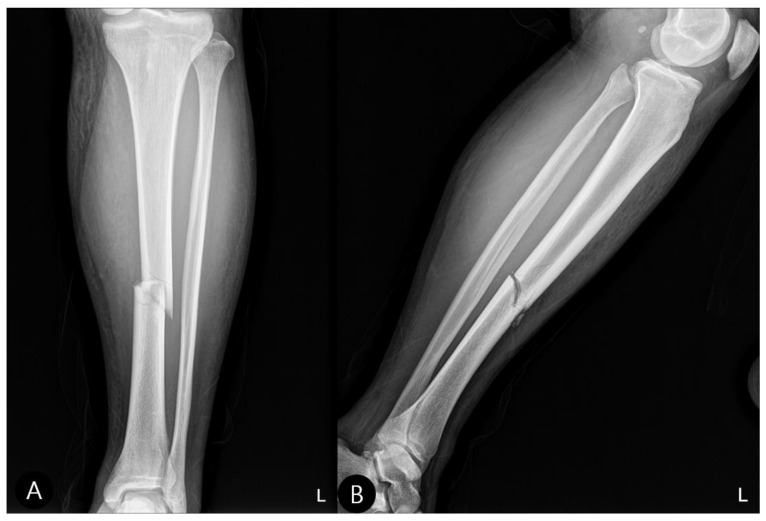
Initial tibia radiographs (2 October 2023). (**A**) Anteroposterior (AP) view showing a displaced fracture of the tibial shaft. (**B**) Lateral view confirming a mid-shaft tibia fracture. “L” indicates the left side.

Initial radiographs confirmed a left posterior hip dislocation with a comminuted fracture of the acetabular posterior wall involving over 30% of the articular surface (Figure 1), alongside a displaced fracture of the left tibial shaft (Figure 2). The location of the acetabular defect was confirmed to be in the non-weight-bearing zone of the posterior wall.

### 2.3. Surgical Technique: Step-by-Step Description

The novel technique is described in detail below. Key modifications from standard ORIF include the acceptance of fragment debridement rather than reduction, the strategic placement of screws to act as a raft, and the precise application of PMMA as an articular surface substitute.

**Figure 3:**
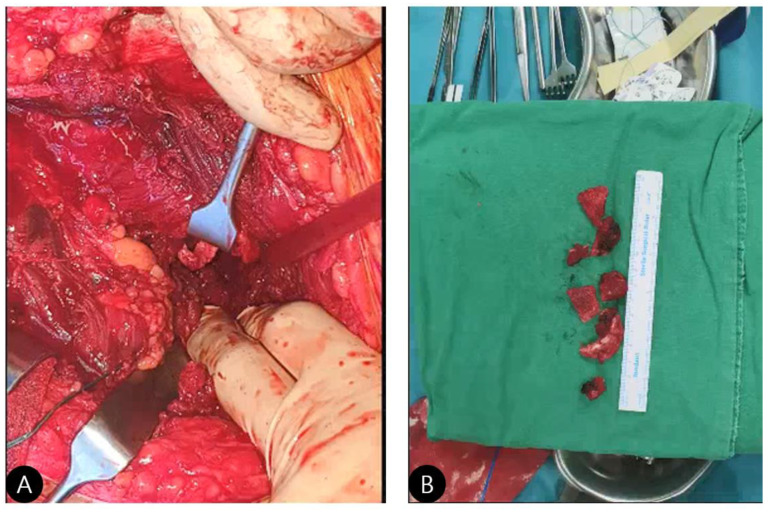
Intraoperative photographs. (**A**) Visualization of bony fragments and posterior subluxation of the femoral head. (**B**) Intraoperative view confirming the presence of six comminuted bony fragments.

**Figure 4:**
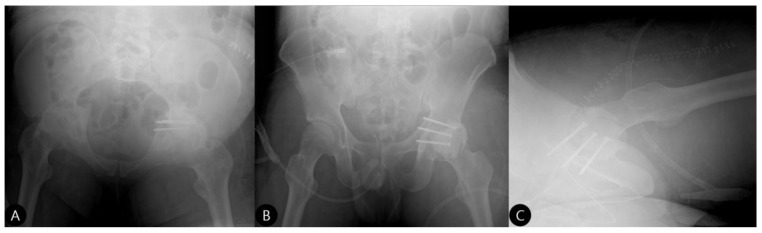
Postoperative pelvic radiographs. (**A**) Outlet, (**B**) inlet, and (**C**) cross-table lateral views demonstrate successful reduction in the hip joint and the position of the cement-augmented construct.

**Figure 5:**
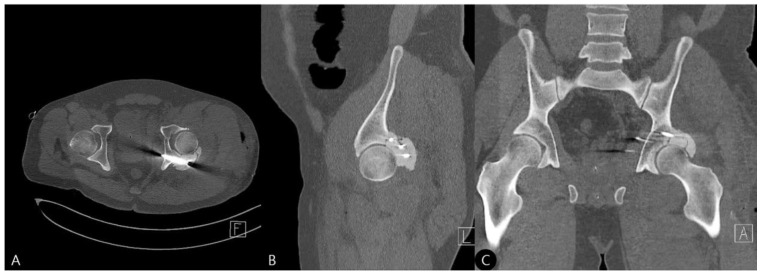
Postoperative computed tomography (CT) scans. (**A**) Axial, (**B**) sagittal, and (**C**) coronal views confirming anatomical reduction in the femoral head, reconstruction of the posterior wall defect, and the relationship between the screw framework and PMMA cement. “F” denotes the feet side, “L” denotes the left side, and “A” denotes the anterior side.

**Figure 6:**
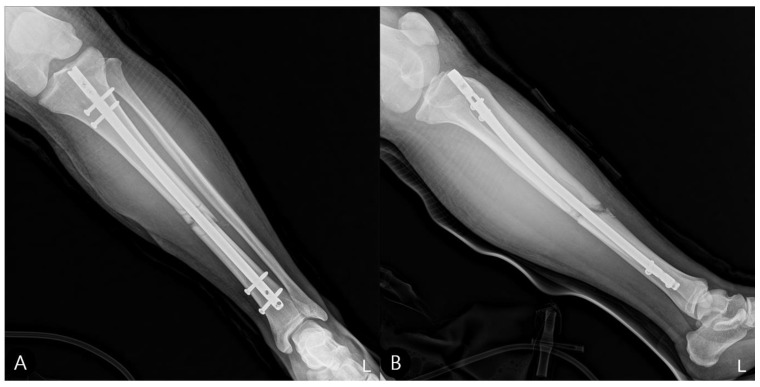
Postoperative tibia radiographs following intramedullary nailing. (**A**) AP and (**B**) lateral views showing satisfactory fracture alignment and stable fixation. “L” indicates the left side.

1. **Positioning and Approach:** The patient was placed in the lateral decubitus position. A standard Kocher–Langenbeck posterolateral approach was utilized, with careful identification and protection of the sciatic nerve throughout the procedure.
2. **Exposure and Assessment:** The hip joint was exposed. The femoral head was found to be dislocated posteriorly. The posterior wall was severely comminuted into six small, non-viable osteochondral fragments (Figure 3), confirming the impossibility of anatomical reduction and stable fragment fixation.
3. **Debridement:** All irreparable, devitalized fracture fragments were meticulously debrided to create a clean, bleeding bone bed for cement interdigitation.
4. **Framework Construction** (**The “Raft”**)**:** Three 3.5 mm cortical screws were inserted from the stable posterior column (ilium) into the remaining stable bone bed of the posterior wall. The key technical point here is that these screws are not placed for interfragmentary compression but to create a stable metallic framework or “raft” [8]. This scaffold provides a mechanical anchor and prevents the cement mantle from displacing under load.
5. **Cement Augmentation (The Critical Step):**
  1. **Material:** Standard surgical-grade PMMA bone cement was used.
  2. **Timing:** The cement was applied in its late doughy phase to minimize the risk of intravasation and allow for manual contouring.
  3. **Application:** The cement was carefully applied over and around the screw heads, meticulously sculpted to recreate the native acetabular curvature and provide a congruent socket for the reduced femoral head.
  4. **Thermal Management:** Continuous saline irrigation was applied over the cement during the exothermic polymerization process to mitigate the risk of thermal necrosis to the surrounding viable bone and cartilage [9,10].
  5. **Precision:** Meticulous care was taken to ensure no cement extended beyond the reconstructed margin into the joint space.
6. **Reduction and Final Assessment:** The hip was reduced under direct vision. Intraoperative fluoroscopy confirmed a concentric reduction, correct implant placement, and the absence of cement fragments in the joint. The stability of the construct was tested through a range of motion. Postoperative imaging confirmed the anatomical reduction and the accurate contouring of the cement construct (Figure 4 and Figure 5).
7. **Concomitant Injury Management:**The tibial shaft fracture was subsequently stabilized with an antegrade intramedullary nail (Figure 6).
8. **Closure:** The wound was closed in layers over a drain.

### 2.4. Fragment Boundary, Cement Volume, and Screw Angle Determination

Fragment boundary determination: The fragment was deemed unreconstructible due to its extremely small size (less than 15 mm in maximum diameter) and poor bone quality that precluded stable screw fixation. The fragment margins showed significant comminution with loss of cortical continuity, making conventional reduction and internal fixation technically unfeasible. Cement volume calculation: Following complete fragment removal, the acetabular defect volume was estimated using intraoperative measurement. Approximately 20–25 mL of polymethylmethacrylate (PMMA) cement was prepared, with the final amount determined by the defect size to achieve adequate fill without overpacking. Screw angle determination: Three screws were placed following anatomical considerations of the acetabular columns. The screw trajectory was planned to achieve optimal purchase in the posterior column while respecting the anatomical angles of the acetabulum, specifically targeting the dense bone of the sciatic buttress and avoiding joint penetration.

### 2.5. Complication Prevention Strategies

Thermal injury prevention: To prevent thermal damage to surrounding tissues and intra-articular structures, we employed continuous saline irrigation during cement polymerization. Additionally, protective padding was placed between the cement and adjacent soft tissues, particularly around neurovascular structures.

Cement extrusion management: The same protective padding and continuous irrigation protocol effectively prevented cement extrusion into undesired locations. The irrigation helped maintain optimal cement consistency and prevented unwanted migration into the joint space or surrounding soft tissues.

Mechanical failure prevention: To address potential mechanical failure, we employed a rafting technique using three screws placed in different anatomical planes of the acetabulum. This multi-directional screw configuration provided superior mechanical stability compared to conventional parallel screw placement.

## 3. Results

### 3.1. Intraoperative Results

The procedure was completed successfully without neurovascular complications. The constructed cement mantle provided immediate stability, allowing for a concentric reduction in the hip joint.

### 3.2. Postoperative Course and Rehabilitation

The patient’s recovery was uneventful. Weight-bearing as tolerated was initiated on postoperative day 3 [11], highlighting a key advantage of this technique’s immediate stability. The patient was discharged on day 7 to begin formal physiotherapy.

### 3.3. Follow-Up Outcomes

**Figure 7:**
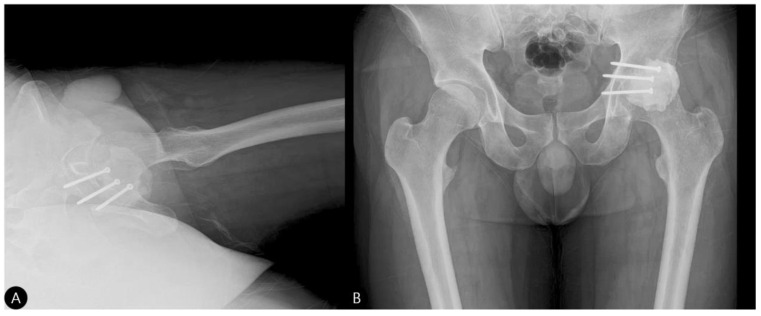
Three-month postoperative follow-up radiographs. (**A**) Cross-table lateral view showing a stable hip joint without subluxation. (**B**) AP view of the hip demonstrating a maintained joint space without evidence of screw migration.

**Figure 8:**
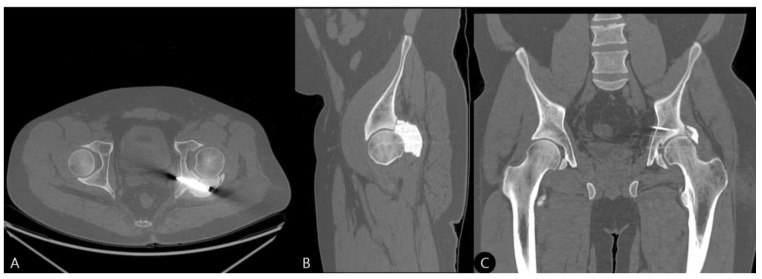
Fifteen-month postoperative follow-up CT scans. (**A**) Axial, (**B**) sagittal, and (**C**) coronal views showing a stable hip joint without signs of subluxation or loosening.

**Figure 9:**
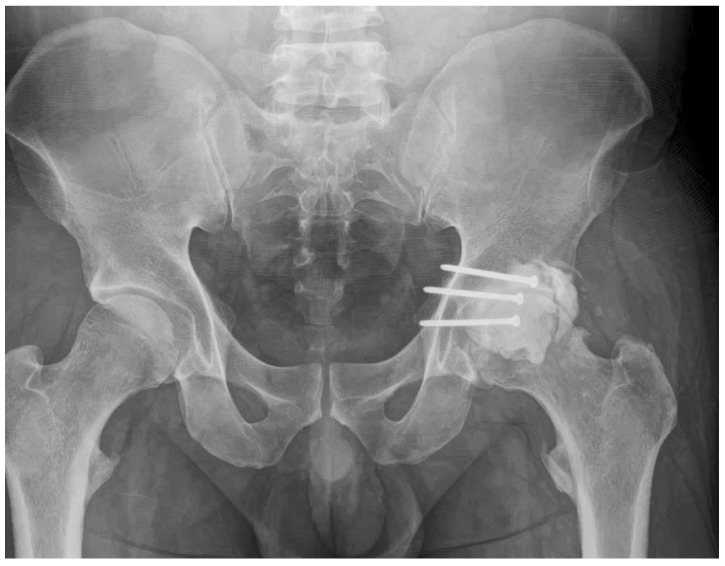
Twenty-one-month postoperative follow-up AP pelvic radiograph. The image shows no evidence of screw loosening and a well-maintained joint space.

- **3 Months:** Radiographs showed a stable hip construct with no signs of subluxation or screw migration (Figure 7). The patient had returned to activities of daily living without major discomfort. The mHHS was 78/100.

- **15 Months:** A CT scan showed a stable joint, concentric reduction, and no signs of screw loosening or osteolysis (Figure 8). The patient reported no functional limitations. The mHHS was 82/100.

- **21 Months:** AP pelvic radiographs showed excellent maintenance of joint space and a stable construct without loosening (Figure 9). The patient’s functional outcome was excellent, reporting no pain during daily activities, with only mild discomfort during deep squatting. The mHHS was 85/100.
- No radiographic evidence of cement loosening, screw migration, or secondary osteoarthritic change was observed at any follow-up interval.
- The mHHS excludes cross-legged sitting ability, making it more appropriate for functional assessment in cases where this specific activity may be limited. All other activities of daily living were performed without difficulty throughout the follow-up period.

## 4. Discussion

### 4.1. Novel Aspects of Our Technique

Traditional management of unreconstructible acetabular posterior wall fractures typically involves primary total hip arthroplasty (THA), which is considered the treatment of choice when anatomical open reduction and internal fixation is not feasible. However, this approach presents significant limitations in young, active patients due to concerns over long-term implant survivorship and the inevitability of revision surgery.

Our technique represents a novel joint-preserving alternative that utilizes cement augmentation after complete fragment removal and strategic screw placement. This approach differs fundamentally from conventional cement augmentation techniques that attempt to preserve comminuted fragments. Instead, we completely remove unreconstructible fragments and use PMMA cement as a structural substitute, creating a stable foundation for screw fixation while preserving the native hip joint. This methodology bridges the gap between conventional ORIF and primary THA, offering a joint-preserving solution for cases previously deemed suitable only for arthroplasty.

The importance of joint-preserving approaches in acetabular fractures has gained increasing recognition, particularly as surgical approach selection significantly affects both complication rates and reduction quality and clinical outcomes [12]. Our technique represents an extension of this philosophy, offering a viable alternative to primary THA in cases previously considered unreconstructible.

This technical note details a novel application of PMMA cement augmentation for a specific, challenging clinical scenario. The technique’s novelty lies not in the use of cement itself, but in its specific application as an articular surface substitute in the acetabulum, supported by a screw raft, for unreconstructible fractures in a non-weight-bearing area.

The management of severely comminuted acetabular posterior wall fractures represents a significant dilemma in orthopedic trauma surgery. While ORIF is the gold standard, it is not always feasible [4,7,13]. Traditional salvage options include biologic reconstruction using autologous or allogeneic bone graft or acute THA [13,14]. However, large structural grafts carry risks of resorption and collapse [14], and primary THA in young patients exchanges the immediate problem for the certainty of future revision surgery [6,15].

The technique described herein—cement augmentation over a screw framework—offers a novel solution. The screws act as a reinforcing scaffold, mitigating the risk of cement fragmentation under load, while the PMMA cement provides immediate stability and recreates a congruent articular surface. This approach preserves precious bone stock, making any future conversion to THA technically simpler [16].

**Key Technical Considerations and Tips for Success:**

- **Strict Indications is Crucial:** This is strictly a salvage procedure for unreconstructible fractures confined to the *non-weight-bearing* portion of the posterior wall. It is contraindicated for weight-bearing dome fractures or when viable fragments can be fixed.
- **Stable Framework:** The primary role of the screws is not interfragmentary compression but to create a stable mechanical anchor for the cement mantle [8].
- **Meticulous Cement Handling:** Apply cement in the late doughy phase to prevent extravasation. Continuous irrigation is essential to reduce thermal necrosis [9,10]. Precise contouring to match the native acetabular curvature is critical for achieving joint congruence.
- **Early Mobilization:** The immediate stability afforded by the cement permits early weight-bearing [11], which is crucial for functional recovery and preventing stiffness.

### 4.2. Long-Term Cement Management Strategy

Our current plan is to maintain the PMMA cement in situ indefinitely unless specific complications arise. This decision is based on several considerations: First, delaying total hip arthroplasty (THA) conversion as long as possible is advantageous in this young patient, as it minimizes the lifetime number of revision procedures. Second, staged cement removal carries significant risks including debris generation, soft tissue adhesion formation, and potential cartilage damage during extraction. The primary risks of long-term cement retention include potential debris generation from mechanical wear, adhesion formation around the cement-bone interface, and possible cartilage damage if cement particles enter the joint space. However, these risks must be weighed against the benefits of joint preservation and the inevitable complications associated with earlier THA conversion in young patients. Should conversion to THA become necessary, the cement can be carefully removed during acetabular preparation, though this may require specialized techniques to minimize bone loss and optimize component fixation.

**Limitations:** The primary limitation is the unknown long-term behavior of the PMMA cement articulating directly with cartilage [17] and the potential for accelerated osteoarthritis. Furthermore, the technique is highly specific to a particular fracture pattern and lacks validation from large-scale comparative studies. Long-term follow-up beyond 21 months is essential to monitor for potential late complications such as wear-induced osteolysis or mechanical failure.

## 5. Conclusions

This technical note provides a detailed description of cement-augmented screw fixation as a novel joint-preserving salvage procedure for young patients with unreconstructible, comminuted fractures of the non-weight-bearing acetabular posterior wall. The technique offers a viable alternative to primary THA by providing immediate stability, enabling rapid rehabilitation, and preserving native bone stock. While long-term outcomes require further investigation, this method represents a promising and technically feasible addition to the armamentarium for managing these complex injuries.

## Data Availability

The original contributions presented in this study are included in the article. Further inquiries can be directed to the corresponding authors.
